# Perspectives on reasons for non-adherence to medication in persons with schizophrenia in Ethiopia: a qualitative study of patients, caregivers and health workers

**DOI:** 10.1186/1471-244X-13-168

**Published:** 2013-06-17

**Authors:** Solomon Teferra, Charlotte Hanlon, Teferra Beyero, Lars Jacobsson, Teshome Shibre

**Affiliations:** 1 Department of Psychiatry, School of Medicine, College of Health Sciences, Addis Ababa University, Addis Ababa, Ethiopia; 2 Division of Psychiatry, Department of Clinical Sciences, Umeå University, Umeå, Sweden; 3 Centre for Global Mental Health, Institute of Psychiatry, King’s College London, London, UK

**Keywords:** Adherence, Antipsychotic, Ethiopia, Medication, Qualitative, Schizophrenia

## Abstract

**Background:**

Levels of non-adherence to antipsychotic medication in persons with schizophrenia in rural African settings have been shown to be comparable to those found in high-income countries. Improved understanding of the underlying reasons will help to inform intervention strategies relevant to the context.

**Methods:**

A qualitative study was conducted among persons with schizophrenia (n = 24), their caregivers (n = 19), research field workers (n = 7) and health workers (n = 1) involved in the ongoing population-based cohort study, ‘The Butajira Study on Course and Outcome of Schizophrenia and Bipolar Disorder’, based in rural Ethiopia. Six focus group discussions and 9 in-depth interviews were conducted to elicit perspectives on non-adherence to antipsychotic medication. Thematic analysis was used to identify prominent perspectives.

**Results:**

Predominant reasons for non-adherence specific to a low-income country setting included inadequate availability of food to counter appetite stimulation and the perceived strength of antipsychotic medications. The vital role of the family or other social support in the absence of a statutory social safety net was emphasised. Expectations of cure, rather than need for continuing care, were reported to contribute to non-adherence in the longer-term.

Many of the factors associated with non-adherence in high-income countries were also considered important in Ethiopia, including lack of insight, failure to improve with treatment, medication side effects, substance abuse, stigma and dissatisfaction with the attitude of the care provider.

**Conclusion:**

This study identifies additional barriers to medication adherence faced by persons with schizophrenia in Ethiopia compared to those in high-income countries. In this era of scaling up of mental health care, greater attention to provision of social and financial assistance will potentially improve adherence and thereby enable patients to benefit more fully from medication.

## Background

The discovery of antipsychotic medications has improved outcomes in persons with schizophrenia through control of symptoms [1,2]. Several reports, including from the population-based cohort of persons with schizophrenia in Ethiopia, have shown that adherence to medication is associated with better clinical outcome and lower mortality [3-5].

In practice, the biggest challenge to the effectiveness of antipsychotic medications has been ensuring treatment adherence, defined as ‘the extent to which a person’s behaviour coincides with the medical advice given’ [6]. Measures of adherence vary greatly from study to study including varying percentage times medication was taken, gap in time when no medication was available and pharmacy based measures, the ‘gold standard’ being Medication Event Monitoring (MEM), with a recorder on pill bottle caps measuring time and date when opened [7]. A comprehensive review of the literature had shown at least half of patients prescribed with antipsychotic medications failed to take them [8]. Similar levels of non-adherence were found in our Ethiopian sample [3,4].

Non-adherence to antipsychotic medication is associated with increased risk of relapse, more frequent hospitalization and poorer quality of life [9-11].

Several studies from high-income countries exploring reasons for non-adherence to antipsychotic medication, including some qualitative studies, reported the most common reasons for non-adherence to be lack of insight, low therapeutic alliance [12,13], the presence of positive symptoms, being male, substance abuse, unemployment and low social functioning [6]. Side effects of antipsychotic medications and their subjective tolerability by patients were also reported as reasons affecting adherence [14,15].

There is a dearth of evidence on non-adherence from low and middle income (LAMI) countries. In a qualitative study from South Africa, persons with mental illness reported that long queues when attending for out-patient appointments, which involved a risk of being physically attacked, was an important reason for their non-adherence [16].

Ethiopia has just launched the first National Mental Health Strategy, committing to scale-up of mental health care across the country [17]. In addition, Ethiopia is a pilot site for the WHO’s mental health Gap Action Programme (mhGAP) [18], implemented in collaboration with the Ministry of Health. Improving accessibility of medication is a critical first step in this setting where fewer than 10% of persons with schizophrenia ever received effective treatment [19], but this needs to be coupled with interventions to maximize adherence.

The cohort of patients with schizophrenia in Butajira has been under follow up for over a decade, and from this cohort study, important new evidence emerged regarding the epidemiology of schizophrenia and clinical course and outcome including mortality in a rural African setting. Antipsychotic medications were found to have significant impact in improving clinical course and outcome as well as reducing premature mortality. Unfortunately, adherence to antipsychotic medications was found to be low; this is the reason that prompted us to undertake the current study.

In this qualitative study, we explored the reasons for low adherence to medication in a rural Ethiopian setting from the perspectives of people with schizophrenia, their caregivers, research field workers and health workers with a view to informing interventions to mitigate the problem.

## Methods

### Setting, study design and participants

The study was conducted in Butajira town and the surrounding rural villages, located 135 km south of Addis Ababa, the capital of Ethiopia. The main livelihoods are trade in urban areas and farming in rural areas. The psychoactive stimulant plant, that, is also widely grown as a cash crop. The area is small, but has diverse climatic and geographical features.

This qualitative study was nested within the cohort study “Course and Outcome of Schizophrenia and Bipolar Disorder in Butajira”. The original study involved ascertainment of persons with schizophrenia or bipolar disorder from a community survey of more than 68,000 people using lay interviewers, key informants and clinician-administered diagnostic interviews [19]. Three hundred and twenty one persons with schizophrenia were identified at baseline and have been followed up for over a decade [19]. This project provided a unique opportunity for the population in Butajira by making modern mental health care accessible via a psychiatric nurse-led out-patient unit located in Butajira general hospital. As a benefit of participating in the project, patients were provided free consultation and medication on a regular basis. Prior to the project, people with severe mental disorders living in the Butajira area would have needed to travel to the only psychiatric hospital in the country, located in the capital city, Addis Ababa, in order to receive care. Indeed, for centuries, traditional and religious healers were the major sources of care for people with mental illnesses in Butajira as is the case in rural Ethiopia in general. Details of how mental disorders are seen by the community and where help was sought in Butajira can be found in a report by Alem et al. [20]. The training of large numbers of specialized mental health nurses has resulted in an expansion of basic mental health services across the country [21] Further scale-up of mental health care is underway by integrating mental health services into primary care and general health care facilities. Outside of the main urban centers, only a limited choice of psychotropic medications is available: first generation antipsychotics (mostly chlorpromazine, and sometimes haloperidol, trifluoperazine and fluphenazine decanoate depot) and older antidepressants (amitriptyline). Medications to alleviate movement side effects of first generation antipsychotic medications (e.g. anticholinergic medication) are rarely available. Psychiatric care is mainly pharmacologic; hence, for the majority of patients with severe mental illnesses in Ethiopia, there is no long-term psychosocial rehabilitation service.

Six focus group discussions (FGDs), composed of 6 to 8 participants, and nine in-depth interviews (IDIs) were conducted involving persons with schizophrenia, their caregivers and health care and research field workers. Of these, three FGDs and four IDIs involved patients, two FGD and four IDIs for caregivers, and one FGD for mental health research field workers who provide community outreach to the patients. One IDI was carried out with a psychiatric nurse in the Butajira hospital psychiatric clinic. We chose to conduct both FGDs and IDIs because these different methods of data collection can yield complementary information. In FGDs, it is possible to see how diverse views are held in relation to one another and how the topic is discussed in front of others. On the other hand, a potential advantage of IDIs is that it may be easier to explore potentially sensitive topics that participants might not feel comfortable to discuss in groups, for example relating to stigma or sexual side effects of medications. As per recommendations for the conduct of FGDs [22], participants within a group were homogenous as far as possible in order to facilitate a free and open discussion and ‘capitalize on shared experiences’ [22].

As previous reports have found differing views regarding factors affecting adherence among patients, caregivers and health professionals [14,23], we included participants representing these three perspectives. Any future intervention to try to improve adherence levels would need to take into account caregiver perspectives given the active decision-making role that families play in the mental health care of an affected family member.

Project field workers identified potential respondents and invited them to participate. Purposive sampling was employed to identify patients who had a history of non-adherence to antipsychotic medications, and later in the process, we also interviewed patients with apparently high levels of adherence in order to understand their perspective and incorporate into the emerging themes.

Non-adherence was defined as failing to take prescribed antipsychotic medication in the previous month as reported by patients and family members. Health professionals also considered those patients missing their clinic appointments as non-adherent, as the clinic was the only way for patients to access medications.

A topic guide was developed by the authors and used to guide exploration of the perceived factors affecting adherence. As data collection progressed, CH met with TB on regular basis to discuss the transcripts and allow iterative development of the topic guide to explore emerging themes in greater depth. The topic guide included questions related to explanatory models of illness (including symptoms, causes, course and appropriate interventions), open questions about the experience of being treated with psychotropic medications and perceived reasons for non-adherence; followed by probing with direct questions about the impact of substance use and the nature of the interaction with mental health workers upon adherence All IDIs and FGDs were conducted by TB, a physician who was head of the research project site and involved in treating patients as well as providing consultation to the psychiatric nurses. The transcripts indicated that most participants appeared comfortable discussing reasons for non-adherence. The IDIs and FGDs took place close to where patients lived and lasted from 30 to 90 minutes.

### Data analysis

The IDIs and FGDs were tape recorded. Hand written notes were also taken when deemed necessary. The audiotapes were transcribed into Amharic, Ethiopia’s official language, and then translated into English by TB. TB, ST and CH read through all of the transcripts. Thematic analysis, the most widely used method in qualitative studies [24], was used to identify prominent perspectives on non-adherence. The theoretical position we took was that of ‘essentialist or realist method, which reports experiences, meanings and the reality of participants’ [24]. Coding was done manually, close-to-the-text, and was thus inductive [24], paying attention to key words that described an idea followed by categorization into themes. We coded patients’, caregivers’, field workers’ and health care worker’s responses separately and explored how each group differed by comparing across the groups of participants. A joint meeting was held to discuss the codes and identified themes. According to Braun and Clarke, ‘a theme captures something important about the data in relation to the research question, and represents some level of patterned response or meaning within the data set.’ [24]. There were no major differences among the authors with regards to the salience of the identified themes.

### Ethical considerations

The study had ethical approval from the Science and Technology Agency of Ethiopia and Faculty of Medicine Research and Publication Committee, Addis Ababa University. Participants were informed about the purpose of the study, their right to refuse to participate without any adverse impact on the care they received, as well as measures to ensure confidentiality. After this explanation, they provided verbal consent.

## Results

Participants identified a number of factors that were considered to affect adherence to antipsychotic medications in persons with schizophrenia. There was a substantial overlap in the responses from the four groups of participants and therefore, for the most part, their perspectives are presented together. Issues that were emphasized by a particular group are presented separately.

### Poverty

The most frequent reason for non-adherence antipsychotic medications mentioned by participants was lack of adequate and proper food. Antipsychotic medications were reported to be ‘strong’ which require ‘good’ food in order to counterbalance their side effects. If taken on an empty stomach, both patient and caregiver participants reported that the medications would cause harm to the body. As a consequence, patients were liable to stop their medications whenever there was a shortage of food. Lack of food was also important from another perspective. Participants reported that the medications led to increased appetite. This was reported to have two negative effects: either a burden on the family because of increased food consumption by the patient or, in the event of limited food availability, the experience of hunger. Many participants spoke of being forced to discontinue their medications because they found the increased hunger difficult to tolerate. A patient participating in one of the groups described it as follows:

“If we had good foods like vitamins, we could have withstood the effect of the drugs. The drugs make you weak especially when they are not taken with proper food. Our minds get clearer, but our bodies are getting weaker.”

The health worker (psychiatry nurse) also agreed with this by stating the reports he heard that medications made patients hungry, and they had to stop it whenever they couldn’t get enough food.

“Yes, there are people who have said they couldn’t tolerate the hunger and stopped taking it [the medication]. I have also met families who made them stop taking the drug because they can’t afford to feed them when the patients take the drug, as it makes them eat a lot. There are families who have told me that they had no resources to feed them. This reason is often cited as a factor for stopping treatment.”

### Lack of support from family

Patients mentioned lack of support from their family as one of the most important reasons for discontinuing treatment and follow up. Indeed, respondents commented that adherence was impossible without the active involvement of the family. Various reasons were given for the decrease in family support observed in some cases, including competing work demands for family members. A participant in a patient’s group said:

“My family used to take care of me when I was sick, but now when I ask them to accompany me here [to the hospital] for the follow up, none of them are cooperative. I have to beg them and plead with them. Even my wife says she has a lot of work to do and can’t come; she doesn’t realize that if I get sick she is also going to suffer.”

### Acute illness perspective and stigma

Participants reported that the need for long-term treatment even in the absence of symptoms was alien, leading patients to discontinue their medication prematurely, as illustrated below by a participant in field worker’s group:

“When I visit them, I ask them why they have stopped taking the medication, and many of them tell me that they stopped taking the medication because they felt better. When they get better and start to work and lead a normal social life, they don’t see the need to continue taking the medication.”

Stigma was mentioned to be a powerful barrier to adherence, especially among those who had improved and were now functioning well. It was reported that this group did not want to be considered mentally ill and did not want to be seen having any contact with mental health professionals. One of the field workers said:

“I have also met people who do not want [other] people to know that they are mentally ill. One, for example, told us not to talk to him in public and has stopped the medication just because he didn’t want others to know that he is mentally ill. There is also another who wants to be given the treatment as soon as he arrives and goes away before meeting anybody. A patient by the name… has told me to just greet him when I meet him in the village, but not talk to him for any length of time or ask him whether he feels well or not.”

### Related to the health care provider

Although not consistent across participants, another recurring reason for non-adherence that was especially emphasized by caregivers, field workers and the health worker related to the mental health service. The attitudes and behaviour of health workers were sometimes reported to be off-putting. Some respondents reported feeling ashamed if they missed one appointment and that this would deter them from attending again because of the risk of a negative response from the health worker. The long waiting times and inflexibility of the appointment system also deterred people from continuing with treatment.

### Clinical factors

#### Side effect of medications

The other most frequently mentioned reason was side-effects of medications. Patients mentioned several distressing side-effects such as ‘paralysis of the body’, twisting of the neck, drooling of saliva, weakness, and daytime sleepiness which interfered with their functioning. The critical impact of appetite stimulation on adherence has already been described in relation to the first theme.

#### Concurrent alcohol and drug use

Alcohol drinking and khat, an amphetamine-like stimulant, chewing were reported to be important factors adversely affecting adherence. Caregivers, field workers and health workers especially emphasized the role of alcohol and khat in affecting adherence. One caregiver said ‘He doesn’t take the medicine; he says it prevents him from chewing khat.’ Sometimes the caregivers themselves would not give patients the medication when they drank alcohol saying alcohol did not agree with the medication. A caregiver reported:

“He says, ‘I have taken alcohol, and therefore, I will not take the tablets.’ He says, ‘It doesn’t suit me, it makes me uncomfortable. He even says ‘I have now finished the tablets, so give me some *araki* [strong locally brewed liquor]’.”

#### Lack of insight

Patients’ lack of awareness of having a mental illness was another barrier that was observed by family members for not adhering to medication. Family members were involved in reminding patients about appointments, bringing patients to the hospital and ensuring that they took the medications. The following quote illustrates the challenges faced by the caregivers.

“We usually urge him [the patient] to go to the hospital for the follow-up for his treatment. We would tell him he would be given money there. He would sometimes become angry with us. They [the health workers] tell us to give him the tablets daily. At first, he agreed to take the tablets himself on his own. He took it from me and put it in his pocket. He sometimes would take it and sometimes miss it, and finish it somehow. But, later on, he would just disappear when his appointment date comes.”

Similarly, according to experiences reported the clinician, lack of insight was among the barriers affecting patients’ willingness to come to the hospital to collect their medications or get their monthly injections and the role of the family as ‘substitute insight’ as illustrated below:

“Because patients have no insight, their insights are their families. Therefore, there are a lot of people who stop taking their medications because of lack of insight.”

## Discussion

In this qualitative study from rural Ethiopia we explored the reasons for non-adherence to antipsychotic medications in persons diagnosed with schizophrenia, from the perspectives of patients, their families, health professionals and research field workers. Several of the reported reasons for non-adherence were in keeping with previous findings from high-income countries [6,8,16], illustrating commonalities across settings. However, the particular contribution of this study was the finding that those factors considered most important to medication adherence were peculiar to this low-income country setting.

Economic factors such as lack of access to adequate nutrition emerged as the most salient factor for non-adherence from the perspective of the respondents. Chronic food insufficiency is still common in the study site [25] despite recent positive developments in the country, and patients with severe mental illnesses were reported to have more chronic energy deficiency compared with their healthy counterparts [26]. In fact, a 5-year mortality report from the same cohort of patients with schizophrenia showed severe malnutrition as the second most common cause of death, accounting for 13.2% of the deaths [4]. In a situation where there is food shortage at the household level, healthy members of the family get the priority.

The inter-relationships between food insufficiency and non-adherence to antipsychotic medication appear to be multi-faceted. Antipsychotic medications are known to increase appetite and craving for food which is generally believed to be mainly through stimulation of the histaminergic neurotransmitter system [27]. Appetite stimulation by the medication would lead to feeling of hunger (subjectively distressing) or increased food consumption (increased burden on family). While there are some studies from western countries, where there is abundance of high fat and high calorie foods, suggesting increased appetite and the consequent weight gain adversely affecting adherence to antipsychotic medications [28], there is paucity of data from low income countries where food is scarce. But, a review of the HIV literature from sub-Saharan African countries indicated lack of food or hunger being an important factor affecting adherence. Patients reported increased appetite caused by the medications, and inability to get adequate food forced them to stop the medications [29,30]. This same experience was reported by our patients as well as their caregivers.

A previous qualitative exploration of purposively selected patients from the Butajira course and outcome study found that one of the justifications patients gave for ongoing chewing of the leaves of khat, an amphetamine-like stimulant which has anorexigenic properties, was as a means to curb their appetite. Family caregivers who couldn’t provide food would allow patients to chew khat despite being aware of its negative impact on the patient’s illness such as worsening of symptoms [31]. However, another factor linked inadequate food availability with non-adherence: perceiving the medication to be ‘strong’, requiring the person to take adequate food in order to be able to tolerate it, emerged as a strong factor affecting adherence. The apparent fatigue which could potentially be caused by the typical, low potency antipsychotic medications (e.g. chlorpromazine and thioridazine) that the patients were taking was perceived to be caused by the lack of a balanced diet [32].

Another important factor reported to affect medication adherence was the role of the family in the care of patients. In the absence of community-based mental health care, patients relied on family to access care. Patients may be too unwell, lack insight or be disabled by negative and cognitive symptoms of schizophrenia, to be able to remember their appointment dates and attend clinics on their own, particularly as health facilities are usually some distance from the patient’s place of residence. As well as enabling the patient to access care, family members also play an important role in ensuring that their affected relative takes their medication, through reminders and encouragement. A recent randomized controlled study carried out in Peshawar, Pakistan, found that adherence significantly improved whenever patients were supervised by relatives, an approach which the authors called STOPS, Supervised Treatment in Out-Patient for Schizophrenia [33]. The actions of family members to promote adherence may go beyond mere encouragement. In this study, some family members went so far as to mix the medication with drinks (tea, milk) covertly, while others employed various coercive techniques to make the patients under their care take the medication. Such covert administration of treatment and coercion is commonly practiced on patients living in LAMI countries [34]. It has been argued that, for severely unwell persons who lack the capacity to make decisions about their treatment, and in the absence of adequate community-based services, such actions may serve to promote, rather than undermine, a patient’s autonomy through restoring their health [35]. However, patients can be abused in the process, in addition to issues relating to autonomy and consent [36].

Exclusive reliance upon family support is also problematic when such support is not consistently present, as was reported to be the case by participants in this study. Caregiver burden, both economic and emotional, may contribute to the waning support over time, although a previous report from this study showed that accessing effective treatment was one of the most important factors in alleviating burden [37]. Without the support of families, many persons with schizophrenia and other severe mental disorders in this setting become vagrants or destitute, contributing to their greatly reduced life expectancy [4].

The other important factor reported to affect adherence was lack of knowledge about the nature of the illness, the medications and the expected outcome from the treatment. Considering apparent absence of symptoms as cure led to discontinuation of treatment. Or on the contrary, they expected too much from the treatment, and when ‘cure’ didn’t happen, they abandoned the medications and sought help from alternative health care such as holy water treatment, ‘*tsebel*’, or consulted traditional/ indigenous healers. In a rural Ethiopian setting where the burden of acute, curable infectious disease is high, patients and caregivers may find it difficult to shift to a chronic disease model of care and accept the need to continue taking medication [38].

Stigma was another factor reported to affect adherence. Having a severe mental illness such as schizophrenia is highly stigmatizing in the area, with low prospects for work or marriage [20]. It is not only the patients, but also their relatives who are stigmatized [39]. This issue was particularly relevant for patients who were no longer suffering from any symptoms. After showing some improvement, patients were liable to decline the offer of continued care and stop their medication. They were not even willing to talk with the mental health field workers or psychiatric nurses whenever they visited them in their villages.

In this study, one of the reported factors affecting adherence to antipsychotic medications was lack of insight. As reported time and again by family members, lack of insight led to refusal to take medications. Lack of insight is one of the hallmarks of psychosis [40]. There are several explanations for lack of insight in schizophrenia including cognitive deficits, denial of symptoms or a lack of adaptive personal narrative understanding of illness [41]. Although we didn’t specifically look into reasons for lack of insight in the study participants, given the diverse nature of their clinical status, the aforementioned reasons could apply to them as well. There is a complex relationship between insight and medication adherence. Several studies have shown that it is probably one of the most important factors affecting adherence and thereby resulting in poorer outcome [12,42-44]. On the contrary, there are reports which showed that patients might continue to take medication, although they do not believe they have mental illness, because of relief of symptoms from taking medications [24,45].

Medication side effects were frequently cited as an important factor leading to non-adherence in this setting. The only antipsychotic medications available to the patients in the study were a very limited selection of first generation antipsychotic medications (FGAs). These FGAs have the potential to cause distressing side effects in a context where medications to counter side effects is erratically available. Several studies on medication adherence identify side effects as a very important factor affecting adherence [14,15,46]. Unfortunately, the hope that second generation medications would have greater tolerability and, therefore, improved adherence has not been borne out in practice [47,48].

The behavior of some of the health care providers was also mentioned as a factor affecting adherence. Several studies have shown that the therapeutic alliance with patients plays an important role in adherence to prescribed treatments [49-51].

Substance abuse was also reported to be a common reason for non-adherence. Patients who chewed khat and drank alcohol had a reportedly low level of adherence to antipsychotic medications. In keeping with these findings, a previous qualitative study nested in the Butajira cohort study also reported that patients did not like the idea of taking khat and medication together because of fears that harm would result from combining two powerful chemicals [31]. Co-morbid substance abuse is known to adversely affect adherence to treatment in patients with schizophrenia in high-income countries, and a similar picture appears to pertain in a rural African setting [8,13].

The main limitation in this study is the broad definition of non-adherence i.e. subjective report by patients of not taking their medication, family members’ report or a report by health professional based on a missed clinic appointment. Not all participants, patients and families mentioned non-adherence as a problem for them personally, which could be related to social desirability bias or fear of retribution because the interviewer was a doctor working in the psychiatry clinic. Without an objective measure of adherence we couldn't evaluate the impact of social desirability. However, we did know their adherence history as documented in the monthly follow-ups by the Butajira study nurses. So, we had contemporaneous, independent indicators of adherence and were not relying solely on self-report.

## Conclusion

In this qualitative study from rural Ethiopia, we identified those factors considered to be most important for adherence as reported by patients, families, research field workers and health workers. Based on these findings, the following recommendations can be made: interventions that address food insecurity have the potential to substantially improve adherence by (1) reducing the suffering associated with medication-induced appetite stimulation (2) alleviating strongly held concerns that medication is too potent to be taken without adequate nutrition, and (3) relieving the economic burden on families thus enabling them to provide the ongoing support. Such an approach is supported by a recent WHO report which identified persons with severe mental disorders as a vulnerable group to be prioritized for aid and development activities in low-income countries [52]. Families of persons with schizophrenia should, therefore, be targeted by the existing ‘Productive Safety Net Programme’ present in chronically food-insecure areas of Ethiopia. Non-governmental organizations can also play a role in supporting the basic needs of patients, for example, for livelihoods and adequate nutrition. In this respect much can be learned from the strategies used to expand the uptake of antiretroviral therapy in persons living with HIV/AIDS in Ethiopia, where similar factors affected adherence. According to the 2012 country progress report, 103,659 poor HIV positive Ethiopians received food support in addition to medication by the end of June 2011 [53]. Other measures include integration of mental health in primary care to ensure access close to where patients live, equip workers with communication skills that will optimize development of a good therapeutic alliance, ongoing psychoeducation to patients and their families, improve management of medication side effects and concurrent misuse of substances.

## Competing interests

The authors declare that they have no competing interests.

## Authors’ contributions

TB carried out the data collection; TB, ST, CH, LJ, and TS participated in the analysis, and ST and CH drafted the manuscript. TS and LJ conceived of the study, and participated in its design and coordination. All authors read and approved the final manuscript.

## Pre-publication history

The pre-publication history for this paper can be accessed here:

http://www.biomedcentral.com/1471-244X/13/168/prepub
